# A Community Program of Integrated Care for Frail Older Adults: +AGIL Barcelona

**DOI:** 10.1007/s12603-019-1244-4

**Published:** 2019-08-30

**Authors:** Laura Mónica Pérez Bazán, M.B. Enfedaque-Montes, M. Cesari, L. Soto-Bagaria, N. Gual, M.P. Burbano, F.J. Tarazona-Santabalbina, R.M. Casas, F. Díaz, E. Martín, A. Gómez, F. Orfila, M. Inzitari

**Affiliations:** 1 Parc Sanitari Pere Virgili, Area of Intermediate Care, Barcelona, Spain; 2 RE-FiT Barcelona research group, Vall d'Hebrón Institute of Research, Barcelona, Spain; 3 Institut Català de la Salut, Gerencia Territorial de Barcelona de Atención Primaria, Barcelona, Spain; 4 Geriatric Unit, Fondazione IRCCS Ca' Granda Ospedale Maggiore Policlinico, Milano, Italy; 5 Department of Clinical Sciences and Community Health, Università di Milano, Milano, Italy; 6 Department of Medicine, Universitat Autònoma de Barcelona, Barcelona, Spain; 7 Hospital Universitario de la Ribera, Valencia, Spain; 8 School of Nursing, Catholic University of Valencia San Vicente Mártir, Valencia, Spain; 9 Department of Internal Medicine, Hospital Clínic, Institut d'Investigacions August Pi i Sunyer (IDIBAPS), University of Barcelona, Barcelona, Spain; 10 Centro de Investigación Biomédica en Red de la Fisiopatología de la Obesidad y Nu-trición (CIBEROBN), Instituto de Salud Carlos III, Madrid, Spain; 11 Primary Healthcare Center Bordeta-Magòria, Institut Català de la Salut, Barcelona, Spain; 12 Institut de Recerca en Atenció Primaria Jordi Gol, Barcelona, Spain; 13 Avinguda de Vallcarca, 169-205, 08023, Barcelona, Spain

**Keywords:** Frailty, integrated care, physical activity, primary care

## Abstract

**Objectives:**

To assess the 3-month impact on physical function of a program for community-dwelling frail older adults, based on the integration of primary care, geriatric medicine, and community resources, implemented in “real life”.

**Design:**

Interventional cohort study.

**Setting:**

Primary care in Barcelona, Spain.

**Participants:**

Individuals aged ≥80 years (n=134), presenting at least one sign of frailty (i.e., slow gait speed, weakness, memory complaints, involuntary weight loss, poor social support). Intervention: After frailty screening by the primary care team, candidates were referred to a geriatric team (geriatrician + physical therapist), who performed a comprehensive geriatric assessment and designed a tailored multidisciplinary intervention in the community, including a) multi-modal physical activity (PA) sessions, b) promotion of adherence to a Mediterranean diet c) health education and d) medication review.

**Measurements:**

Participants were assessed based on a comprehensive geriatric assessment including physical performance (Short Physical Performance Battery -SPPB- and gait speed), at baseline and at a three month follow-up.

**Results:**

A total of 112 (83.6%) participants (mean age=80.8 years, 67.9% women) were included in this research. Despite being independent in daily life, participants' physical performance was impaired (SPPB=7.5, SD=2.1, gait speed=0.71, SD=0.20 m/sec). After three months, 90.2% of participants completed ≥7.5 physical activity sessions. The mean improvements were +1.47 (SD 1.64) points (p<0.001) for SPPB, +0.08 (SD 0.13) m/sec (p<0.001) for gait speed, −5.5 (SD 12.10) sec (p<0.001) for chair stand test, and 53% (p<0.001) improved their balance. Results remained substantially unchanged after stratifying the analyses according to the severity of frailty.

**Conclusions:**

Our results suggested that a “real-world” multidisciplinary intervention, integrating primary care, geriatric care, and community services may improve physical function, a marker of frailty, within 3 months. Further studies are needed to address the long-term impact and scalability of this implementation program.

## Introduction

Frailty, a dynamic state of decreased biological reserves and vulnerability to stressors, increases the risk of negative health events, such as a rapid progression towards disability, falls, fractures, institutionalization, and death (1). Frailty is potentially reversible (2, 3). Therefore, its early assessment, recognition and management represent an important component of preventive strategies within healthcare systems as well as a challenge (4).

Various studies have shown that interventions aimed to detect at an early stage and manage frailty are effective (2, 5, 6, 7, 8, 9, 10, 11). These interventions incorporate individualized actions aimed to reduce or slow down the progression of frailty towards disability and, consequently, to reduce and/ or eliminate the potential negative consequences on health. Due to the multifactorial nature of frailty, interventions targeting frailty in the community should also be multifactorial, and designed according to the results of a comprehensive geriatric assessment (CGA) when possible [5–9, 12, 13]. Among other interventions, the promotion of physical activity is crucial (14). Such programs may find an ideal place in primary care settings, integrating the expertise of a geriatric team (15). Different studies have approached frailty in the primary care or community setting (5, 6, 7, 8, 9, 13), but most of them had rigidly controlled experimental conditions and schemes and their submission to specific funds make the subsequent sustainability, implementation and scaling-up of the same activities in real life, difficult (5, 7, 8, 9). Under these circumstances, we recently proposed different recommendations to design and develop complex pragmatic interventions aimed at comprehensively managing frailty (16). In agreement with these recommendations, we have designed a multifactorial person-centered intervention against frailty (16).

The aim of the present study is to assess a 3-month impact on physical function of a “real life” program for frail older adults in the community, based on the integration of primary care, geriatric medicine, and community services.

## Methods

The methodology of the +AGIL Barcelona program (“Atenció primària i Geriatria Integrades amb visió Longitudinal” in Catalan) is described elsewhere (16). Shortly, it stems from a strategic alliance between the principal public provider of primary care in Barcelona (Institut Català de la Salut, Àmbit d'Atenció Primària de Barcelona) and the major public provider of intermediate care, geriatrics and palliative care in Catalonia (Parc Sanitari Pere Virgili). The program is conducted in the primary care center of Bordeta-Magòria (Barcelona, Spain), which provides healthcare services to 32,340 citizens (20% aged ≥65 years, 6.9%≥80 years). The protocol was approved by the Clinical Research Ethics Committee of the Institut Universitari d'Investigació en Atención Primaria, Jordi Gol.

+AGIL Barcelona began in July 2016 as an additional service and as a result, as part of the geriatric outpatient clinic which was transferred to the community. The geriatric team, including a geriatrician and a physical therapist, attends the primary care center once a week in order to assess the patients referred by the primary care team (i.e., family physicians, nurses, social workers). The program also fosters the subsequent maintenance of physical activity through the existing community resources. Together with the nearby civic center we co-designed a continuation activity which initially is performed under the supervision of a trainer and progressively shifts towards self-practice. The implementation includes refinement with end-users (both professional and participants) (16).

### Study population

Primary care is in charge of identifying the potential beneficiaries of the intervention. The screening strategy is generally directed to older adults without physical disability and/or acute clinical disease, aged ≥80 years and presenting at least one sign of frailty (i.e., slow gait speed, weakness, memory complaints, involuntary weight loss, or poor social support). In particular, the Gerontopôle Frailty Screening Tool (GFST), an 8-item questionnaire validated for frailty screening in primary care (17), was selected by a primary care team to support the identification of possible candidates. Being this is a “real life” program, different from a rigid clinical trial, the referral is flexible depending on the judgment of the primary care team and eventually include individuals with a higher degree of frailty (i.e. with some already established disability) or “less than frail” (mostly sedentary), as previously detailed (16).

### Assessment

The intervention follows the principles of the Frailty Intervention Trial (9), being tailored according to the problems detected through a CGA conducted by the geriatrician and the physical therapist. Socio-demographic data (age, sex, marital status, living alone), clinical characteristics (past medical history, Charlson comorbidity index (18), and current treatment), functional status (Barthel index for basic [0–100 points] (19) and Lawton index for instrumental activities for daily living [0–8 points] (20)), nutrition (Mini Nutritional Assessment-Short Form [MNA-SF®] (21) plus a standardized assessment of adherence to a Mediterranean diet (22)), cognition (Mini-cog® (23)), depressive symptoms (Geriatric Depression Scale (24)), and physical function (Short Physical Performance Battery [SPPB, 0–12 points] (25)) were collected. Frailty status was measured using the Clinical Frailty Scale (CFS) (26).

### Intervention

The participant and their family or caregiver received the results of the CGA the same day of the evaluation. Through a shared decision-making process, carried out on the basis of motivational interviewing techniques, the geriatrician proposed a tailored strategy to achieve shared goals. The participants retained a copy of the agreements, set goals, and recommendations in plain language. Primary care staff could join geriatric visits and had access to the complete report through the shared electronic health records (EHR).

The multifactorial intervention, as previously described (16) included:

• Physical activity. After a first individual assessment, an expert physical therapist recommended an adapted exercise program to be performed at home, which included strength, resistance, balance, and coordination exercises. The therapist ran a supervised program including up to 10 sessions (1 h/ week) of multi-modal group exercises, where group socialization was also promoted. The therapist modulated the exercises according to each participant's capacity and needs, in terms of intensity, time and type (including dual-tasking if needed). The exercises had a functional and significant character in order to increase the therapeutic adherence and make the exercises similar to the daily activity of the participant. Different strategies were directed to empower the person and to foster adherence to physical activity over time, including the delegation of the participant to lead their last group session for a short time, the recommendation of personalized exercises as “homework” and the indication of existing community resources to maintain physical activity after the 10 sessions. The Vivifrail® app or a printed chart of tailored exercises derived from it, is given to each participant as complementary material (27), and with positive reinforcement based on motivational interviewing.
• Nutrition. The intervention aimed at promoting adherence to a Mediterranean diet, following the Prevention with Mediterranean diet (PREDIMED) intervention paradigm (28), and took into account the caloric and protein intake needs.
• Health education. This included the promotion of healthy habits, reinforcing ongoing activities in the primary care center (e.g., smoke or alcohol control) or others (e.g., sleep hygiene, fall prevention recommendations).
• Adequacy of pharmacological treatment. In agreement with the family physician, the geriatrician reviewed the patients' medication to increase value-based prescriptions. No standardized tool to test the medication adequacy was used owing to the presence of a geriatrician with specific expertise on medication review and de-prescribing. The geriatrician was also remotely supported, in case of need, by the clinical pharmacist working at the intermediate care hospital.

Both the therapist (on a weekly basis, when running the group activity) and the geriatrician (in one single follow-up visit at 3 months) monitored the intervention plan over time. Subsequently, the patient kept the usual follow-up with the referent primary care professional, who continued promoting the achievement of the initially shared goals. The geriatrician's intervention remained on demand. The coordination/interaction of the primary care and geriatric teams was facilitated by a shared EHR and formal meetings on regular basis.

### Outcomes

The primary outcome of interest was the 3-month, pre/post intervention modification of physical performance (measured by either SPPB and/or its sub-item gait speed). As a secondary outcome, the change in the CFS was explored.

### Statistical analyses

Baseline characteristics of the sample are presented as mean values and standard deviation (SD) for continuous variables, median values and interquartile range (IQR) for ordinal variables and percentages for categorical variables. Differences among participants included in the intervention and those who were missing, were analyzed using the Student's t-test or the Mann-Whitney U-test and Chi-square test, as appropriated.

The pre/post intervention analysis was done by a paired sample t-test for repeated samples or Wilcoxon signed-rank for continuous variables and Chi-square for categorical variables. For those participants who were unable to perform the chair stand test (n=9), the value of 61 seconds was imputed according to the reference categories previously published (25). The Analysis of variance (ANOVA) and Jonckheere-Terpstra trend test were used for continuous variables when the analyses were stratified by the frailty degree according to 3 CFS categories (“Managing well or fit”, “vulnerable”, “any degree of frailty”).

A sensitivity analysis was also performed considering the participants who did not undergo the follow-up assessment because of incident events, or for other reasons that could not be ascertained. In these cases, the worst change of the assessed group for either SPPB (3 points loss), gait speed (0.32 m/sec loss), chair stand test (45.6 sec increase) or balance impairment (an impairment at follow-up) were imputed.

Finally, changes in the CFS between baseline and three-month follow-ups were analyzed by the Chi-square test.

In all analyses, p-values <0.05 were regarded as statistically significant. The 95% confidence intervals (95%CI) were calculated. Analyses were performed using Stata version 14.

## Results

**Figure 1:**
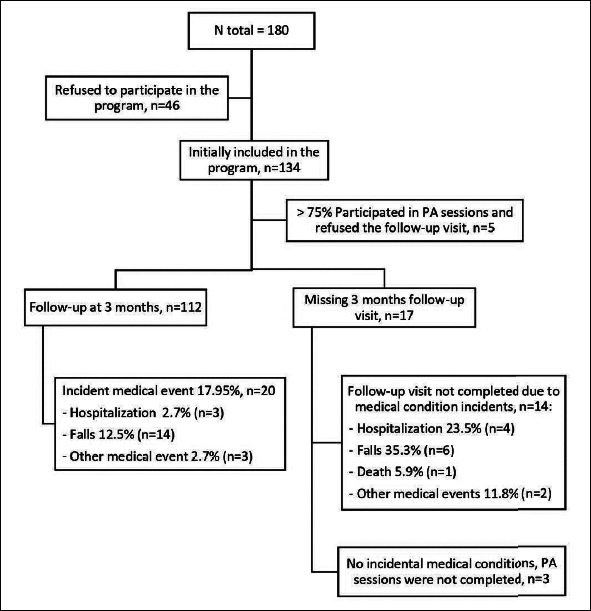
Population follow chart PA: Physical activity.

From July 2016 to July 2018, 134 individuals out of 180 screened (74.4 %) accepted to participate in the program (Fig 1). Twenty-two (16.4%) missed the follow-up (5 refused to undergo the follow-up visit after correctly completing ≥75% of physical activity sessions and without any incident event, 14 because of an intercurrent medical event, and 3 did not attend the follow-up visit despite the absence of medical complications). Finally, 112 individuals (mean [SD] age=80.8 [SD 5.8] years; 67.9% women) were included in the analysis. Baseline characteristics were comparable between the 112 included participants and the 17 excluded because of events or unknown reasons (Table 1).

**Table 1:** Baseline characteristics of the sample

**Baseline characteristics**	**Included n= 112**	**Missing n=17**	**p**
Age, mean (SD)	80.9 (5.8)	82.4 (4.1)	0.210
Woman, % (n)	67.9 (76)	88.2 (15)	0.086
Barthel index ^a^, median (IQR)	95 (90–100)	95 (85–100)	0.494
Lawton index ^b^, median (IQR)	6 (4–8)	7 (4–8)	0.617
Lives alone, % (n)	42.0 (47)	64.7 (11)	0.079
Education, % (n)			
Illiterate	4.5 (5)	12.5 (2)	0.131
Primary school	32.1 (36)	50.0 (8)	
Secondary school	49.1 (55)	37.5 (6)	
University degree	14.3 (16)	0 (0)	
Adequate physical activity ^c^, % (n)	38.4 (43)	23.5 (4)	0.235
Falls in the last year, % (n)	34.8 (39)	41.2 (7)	0.610
Malnutrition risk ^d^, % (n)			
Normal nutrition status	61.6 (69)	76.5 (13)	0.209
At risk of malnutrition	36.6 (41)	17.7 (3)	
Malnourished	1.8 (2)	5.8 (1)	
Charlson Comorbidity, median (IQR)	1 (0–2)	2 (1–4)	0.052
Previous diagnosis of cognitive impairment, % (n)	18.8 (21)	25.0 (4)	0.483
Number of drugs, median (IQR)	7 (5–9)	9 (6–10)	0.171
Gerontopôle FST positive, % (n)	94.6 (106)	100 (17)	0.328
Clinical Frail Scale - vulnerable or any frailty degree, % (n)	63.4 (71)	64.7 (11)	0.917
SPPB ^e^, mean (SD)	7.48 (2.12)	7.18 (2.60)	0.650
Gait speed, mean (SD)	0.71 (0.20)	0.67 (0.20)	0.486
Chair stand test, mean (SD)	22.40 (13.67)	24.52 (18.20)	0.898
Balance impairment, % (n)	43.8 (49)	58.8 (10)	0.245
Physical activity sessions, mean (SD)	9.2 (1.9)	5.4 (3.1)	<0.001


IQR: interquartile range, SD: standard deviation. Student's t-test or the Mann-Whitney U-test were used for continuous variables as appropriated and Chi-square test for categorical. a. Independence for activities of daily living, range from 0–100. b. Independence for instrumental activities of daily living, range from 0–8. c. At least 30 min of physical activity 3 times/ week (based on WHO recommendation). d. Mini-Nutritional Assessment Short form score: 8–11 points. e. Short Physical Performance Battery, range from 0–14

The 112 included participants were generally independent for basic Activities of Daily Living (ADL, median Barthel Index=95, IQR=90–100) and instrumental ADL (IADL, median Lawton's score=6, IQR=4–8), had a relatively low comorbidity (median Charlson index=1, IQR=0–2), but presented polypharmacy (median number of drugs=7, IQR=5–9) and low physical performance (SPPB mean=7.5 [SD 2.1] and gait speed mean=0.7 [SD 0.2]).

Regarding the intervention, physical activity was performed in the whole sample, with a high adherence (90.2% attended ≥75% session, mean [SD]=9.2 [SD 1.9] out of 10 planned sessions), as well as health education interventions and nutritional recommendations, whereas pharmacological treatment was modified in 76 (67.9%) participants.

Regarding the primary outcomes, after three months, the SPPB and gait speed improvements were +1.47 [SD 1.64] points (p<0.001) and +0.08 [SD 0.13] m/sec (p<0.001) respective, as is shown in Table 2. Improvement in chair stand test (−5.5 [SD 12.1], p<0.001), and balance (53%, p<0.001) were also analyzed. Results remained substantially unchanged after stratifying the analyses by frailty degree (Table 3). However, no linear association between improvement and frailty degrees were found. Additionally, after imputing the worst observed changes in the 17 participants without the 3 month visit because of events or for unknown reasons, we confirmed statistically significant improvement in SPPB (+0.88 [SD 2.16] points, p<0.001), chair stand (−10.83 [SD 17.7] sec, p<0.001), balance (44.1% improved, p<0.001), and a nonsignificant improvement in gait speed (+0.02 [SD 0.20] sec, p=0.301).

**Table 2:** Effect of the Multifactorial Intervention on physical function capacity

	**Before**	**After**	**Difference (95%CI)**	**p**
SPPB ^a^, mean (SD)	7.48 (2.12)	8.94 (2.03)	1.47 (1.16 – 1.78)	<0.001
Gait speed (m/sec), mean (SD)	0.71 (0.20)	0.79 (0.18)	0.08 (0.05 – 0.10)	<0.001
Balance impairment, % (n)	43.8 (49)	20.5 (23)	53.1 (26)	<0.001
Chair stand test (sec), mean (SD)	22.40 (13.63)	17.00 (10.62)	-5.50 (−7.78 – −3.22)	<0.001


SD: standard deviation; a. SPPB: Short Physical Performance Batteiy, range from 0 to 12; 90.2% of participants attended ≥75% session, mean (SD)=9.2 (1.9) sessions; For those participants unable to perform the chair stand test (n=9), we imputed a value of 61 seconds according to the reference categories previously published (25); Paired sample t-test for repeated samples or Wilcoxon signed-rank were used for continuous variables as appropriated and chi square for categorical variables.

**Table 3:** Effect of the Multifactorial Intervention on physical function capacity stratified by a frailty degree according to the Clinical Frailty Scale

	**Fit, Stable, managing well (CFS 1–3), n=41**					**Vulnerable (CFS 4–6), n=48**			**Any grade of Frailty (CFS 7–9), n=23**				**p for trend**
	**Before**	**After**	**Difference (95% IC)**	**p**	**Before**	**After**	**Difference (95% IC)**	**p**	**Before**	**After**	**Difference (95% IC)**	**p**	
SPPB a, mean (SD)	8.43	9.68	1.25	<0.001	7.13	8.67	1.54	<0.001	6.52	8.21	1.70	<0.001	0.279
	(2.05)	(2.08)	(0.81 – 1.69)		(2.02)	(1.95)	(.99 – 2.09)		(1.90)	(1.76)	(1.04 – 2.35)		
Gait speed (m/sec), mean (SD)	0.77	0.86	0.08	<0.001	0.71	0.78	0.07	0.005	0.60	0.69	0.09	<0.001	0.940
	(0.18)	(0.17)	(0.05 – 0.12)		(0.21)	(0.17)	(0.02 – 0.11)		(0.14)	(0.16)	(0.04 – 0.13)		
Balance impairment, % (n)	31.7	14.6	53.8	0.001	52.1	25.0	52.0	0.009	47.8	21.7	54.5	0.043	0.540
	(13)	(6)	(7)		(25)	(12)	(13)		(11)	(5)	(6)		
Chair stand test (sec), mean (SD)	18.28	13.97	−4.32	0.002	25.58	18.16	−7.43	<0.001	23.37	19.83	−3.55	0.235	0.352
	(9.99)	(4.85)	(−6.89 – −1.75)		(15.91)	(11.91)	(−11.45 – −3.41)		(13.92)	(13.84)	(−9.56 – −2.47)		


SD: standard deviation; a. SPPB: Short Physical Performance Battery, range from 0 to 12; The Analysis of variance (ANOVA) and Jonckheere-Terpstra trend test were used for continuous variables as appropriated and Chi-Square for categorical variables.

Analyzing the change of frailty degree at 3 months, according to the CFS, 14.3% of the participants experienced progression towards a higher level of frailty, 64.3% remained stable, and 21.43% improved of some degree (p<0.001).

## Discussion

Our results show that +AGIL Barcelona, a multifactorial intervention program for older community-dwellers, based on the integration of primary care, geriatric medicine and community services, has a positive impact on improving frailty. The reported improvement of physical function was statistically and clinically significant, as meeting well-established criteria for meaningful changes in SPPB (0.3–0.8 points) (29) and gait speed (at least 0.05 m/s) [30]. The benefits were consistent across different initial frailty degrees, from milder to more advanced.

Strong clinical evidence from intervention studies support the positive impact of multifactorial and multidisciplinary program interventions on frailty in order to improve physical function (5, 7, 8, 9, 11) and revert frailty (10). In this sense, our results are in line with the available literature. Specifically, +AGIL Barcelona effect size at 3 months is similar to a local study that offered higher physical activity intensity (7) and greater than those reported in a systematic review that analyzes the effect of physical activity on SPPB and gait speed in older adults (14).

Despite the similarities in the multidisciplinary and multifactorial approach, with a common component such as the promotion of physical activity, there are differences and novelties worth noting in +AGIL Barcelona. First, it was and is still implemented in real life and did not depend on specific research funding. In other words, it broke the rigid structure of traditional research methodology concerning the inclusion/ exclusion criteria. For example, dementia or severe frailty did not constitute reasons for being excluded from participation in +AGIL Barcelona (5, 7, 8, 9, 10, 11). In fact, the screening and referral of +AGIL Barcelona relied much on the clinical judgment of primary care teams, as requested by the GFST (17). It is believed that the complementary approach between the continuum of care offered by primary care and the time-limited specialized geriatric intervention represents an added value for the program. Second, in line with the principles of the Frailty Intervention Trial described by Cameron et.al., (9) +AGIL Barcelona proposed a flexible and adaptable intervention within a definite range of possibilities, based on shared decision-making, and far from a “one-fits-all” approach or rigid standard intervention. This might have promoted the adequacy of the intervention and might very well reduce overtreatment (12). The contribution of specialized professionals such as the geriatrician and the physical therapist favors the person-centered adaptation. Third, the promotion of physical activity mainly aimed at fostering user's empowerment, resulted in the intervention becoming sustainable over the long term. We speculate this might be the justification for the similar 3-month impact of +AGIL Barcelona compared to existing literature (5, 7, 9). Despite the resemblance with the other intervention components, +AGIL Barcelona offers a relatively lower frequency and duration of supervised physical activity (60-minute sessions, once a week for 10 weeks). In the aim of the project, the different empowerment strategies (including the motivational approach, the recommendations for self-practice through a validated existing tool such as ViviFrail® (27), the delegation to the participant during the sessions) are meant to compensate the reduced frequency and duration of directed physical activity.

It is important to remark that +AGIL Barcelona is sustainable through a reorganization of existing resources. During the refinement phase of the program, we also requested feedback from participants about the possible lack of adherence to the different interventions, including a higher weekly frequency of the physical activity program. The relatively reduced frequency, together with other factors (such as the “prescription” by reference primary care and the motivational approach) might explain the high adherence to the proposed activities compared to other programs (5, 7, 8, 9).

According to the realistic theoretical framework of the reasons why frailty interventions may or may not work, recently proposed by Gwyther et al. (12), interventions need to be co-designed, multicomponent, include physical activity in a group setting that promotes social interaction and psychological techniques in order to perform a person-centered care intervention which promotes lifestyle changes and patient empowerment. We had highlighted very similar requirements in our previous methodological paper (16), and +AGIL Barcelona seems in line with most of it.

Among the strengths of our study, +AGIL Barcelona is a “real world” intervention program with a continuative implementation, and we have already highlighted the positive differential elements and the potential advantages compared to previous intervention studies. The relatively low number of missing data at the follow-up is also remarkable. We also acknowledge different limitations in our study: the absence of a control group and the lack of randomization do not allow conclusive evidence on the impact of this intervention. However, building on existing evidence, the study stems from a spontaneous change occurred in our clinical practice, shifting from the classical approach towards a new integrated care model. Second, the follow-up period is relatively short. However, potential maintenance or improvement in physical function even at 3 months, in such an older and frail population, is already a relevant achievement for the individual and for society.

In conclusion, +AGIL Barcelona shows the implementation and evaluation of a multifactorial, interdisciplinary, and integrated care program for frail older adults, which is based on the reorganization of existing resources. This data suggests potential for scaling-up and replicating similar initiatives in other areas, after contextualization with local specificities. From a general perspective, our results reinforce the urgent need of shifting towards a change in paradigms for the management of frailty before the establishment of the disabling process.
